# Pesticides Are Involved With Population Declines of Amphibians in the California Sierra Nevadas

**DOI:** 10.1100/tsw.2001.36

**Published:** 2001-05-01

**Authors:** Donald W. Sparling, Gary Fellers, Laura McConnell

**Affiliations:** USGS Patuxent Wildlife Research Center, Laurel, MD 20708-4017, USA

**Keywords:** pesticides, amphibian population declines, cholinesterase, California, Hyla regilla

## Abstract

Several species of frogs and toads are in serious decline in the Sierra Nevada Mountains of California. These species include the threatened red-legged frog (Rana aurora), foothill yellow-legged frog (R. boylii), mountain yellow-legged frog (R. muscosa), Cascades frog (Rana cascadae), western toad (Bufo boreas) and Yosemite toad (B. canorus). For many of these species current distributions are down to 10% of historical ranges [1,2]. Several factors including introduced predators [3,4,5], habitat loss [2], and ultraviolet radiation [6] have been suggested as causes of these declines. Another probable cause is air-borne pesticides from the Central Valley of California. The Central Valley, especially the San Joaquin Valley, is a major agricultural region where millions of pounds of active ingredient pesticides are applied each year (http://www.cdpr.ca.gov/dprdatabase.htm). Prevailing westerly winds from the Pacific Coast transport these pesticides into the Sierras [7,8].

A study coauthored by scientists from the U.S. Geological Survey and the U.S. Department of Agriculture and currently in press in *Environmental Toxicology and Chemistry* (http://etc.allenpress.com), shows that amphibians in the Sierra, even in National Parks, are being exposed to these pesticides and that this exposure is causing neurological effects.

Spurred by a growing concern for worldwide declines in amphibian populations, recent research has documented severe declines in North and Central America, Australia, Europe, and Asia and postulated many causes [9]. Whereas contaminants have frequently been suggested as one of the causes for these declines, the effects of pesticides in amphibians is poorly known compared to those in fish, mammals, and birds and this paper is the first to show a clear relationship between pesticides and a regional decline in amphibian populations. Because of the importance of pesticides in protecting crops, the paper also raises potential controversy between Central Valley agriculture and the preservation of amphibian species many miles distant.

The study shows that Pacific treefrogs (*Hyla regilla)* collected from Sequoia and Yosemite National Parks and the Lake Tahoe Basin had lower whole body cholinesterase activity (ChE) than those from coastal reference sites ($p = 0.0001$) and that those at Sequoia NP, downwind from the San Joaquin Valley, had lower ChE than those at Lake Tahoe, downwind of the less intensely agricultural Sacramento Valley. Pacific treefrog served as a sentinel species for the other anurans because their tadpoles occupy the same habitats and there is less concern about collecting treefrogs for analyses than for the less abundant frogs and toads. Cholinesterase is critical to normal neural functioning because it deactivates the neurotransmitter acetylcholine at synapses between neurons and prevents uncontrolled firing of neurons. The most widely used agricultural insecticides, classified as carbamates and organophosphates, function by inhibiting ChE and result in neurological problems typically causing death through respiratory failure. A significant difference in mean ChE activity also occurred when sites were regrouped by ranid population status ($p = 0.0001$); sites with good populations had higher values than those classified as moderate or poor. A ChE depression of 50% or more from a reference value generally serves as forensic evidence of pesticide exposure and 60% of the sample from Sequoia NP had ChE that was below this threshold compared to only 9% of the coastal and 17% of the Lake Tahoe samples with this degree of depression. To further confirm pesticide exposure, subsamples of adult and tadpole treefrogs were analyzed for body burdens of key pesticides. Compared to coastal sites, tissues of tadpoles collected from Sequoia NP had marked increases in the concentration of chlorpyrifos, and those from Lake Tahoe had elevated concentrations of diazinon, two very highly toxic insecticides. More than 50% of the sample at Yosemite NP had measurable levels of either of these two insecticides. Other pesticides which were found at higher concentrations in treefrogs at Sequoia and Yosemite NP compared to reference sites included endosulfan, DDT, DDE, and lindane. Although these pesticides do not affect ChE activity, endosulfan is very highly toxic. DDT, DDE, and lindane are very persistent and highly toxic and probably indicate historic differences in pesticide deposition.

Other studies have demonstrated that pesticides are being carried into pristine National Parks and montane areas in California [7,8]. This study shows that these pesticides are entering amphibians and affecting their nervous systems. Additional research is underway to determine what role these pesticides are having on the survival of amphibians and their population status in California. In other parts of the world many of the most threatened populations of amphibians are in high elevation sites with agriculture and other sources of pollution down slope and downwind. It is possible that these populations may be similarly affected by pesticides and other contaminants [10].
